# Social risk factors for SARS-CoV-2 acquisition in university students: cross sectional survey

**DOI:** 10.1017/S0950268822001698

**Published:** 2022-11-04

**Authors:** Eleanor Blakey, Lucy Reeve, Neville Q. Verlander, David Edwards, David Wyllie, Mark Reacher

**Affiliations:** 1 UK Health Security Agency, East of England Field Service, Cambridge Institute of Public Health, Robinson Way, Cambridgeshire, CB2 0SR, UK; 2 Modelling and Economics Department, UK Health Security Agency, Statistics, 61 Colindale Ave, London, NW9 5EQ, UK; 3 UK Health Security Agency, East of England Health Protection Team, The Mildenhall Civic Hub, Sheldrick Way, Mildenhall, Bury St Edmunds, Suffolk, IP28 7JX, UK; 4 East of England, National Mycobacterial Reference Service South, Cambridge, UK

**Keywords:** COVID-19, infectious disease control, infectious disease epidemiology, surveillance

## Abstract

The objectives of this study were to define risk factors for severe acute respiratory syndrome coronavirus 2 (SARS-CoV-2) infection in University of Cambridge (UoC) students during a period of increased incidence in October and November 2020. The study design was a survey.

Routine public health surveillance identified an increase in the numbers of UoC students with confirmed SARS-CoV-2 positivity in the 10 days after a national lockdown was announced in the UK on 5th November 2020. Cases were identified both through symptom-triggered testing and a universal asymptomatic testing programme. An online questionnaire was sent to all UoC students on 25 November to investigate risk factors for testing positive in the period after 30th October 2020. This asked about symptoms, SARS-CoV-2 test results, aspects of university life, and attendance at social events in the week prior to lockdown. Univariate and multivariable analyses were undertaken evaluating potential risk factors for SARS-CoV-2 positivity.

Among 3980 students responding to the questionnaire, 99 (2.5%) reported testing SARS-CoV-2 positive in the period studied; 28 (28%) were asymptomatic. We found strong independent associations with SARS-CoV-2 positivity and attendance at two social settings in the City of Cambridge (adjusted odds ratio favouring disease 13.0 (95% CI 6.2–26.9) and 14.2 (95% CI 2.9–70)), with weaker evidence of association with three further social settings. By contrast, we did not observe strong independent associations between disease risk and accommodation type or attendance at a range of activities associated with the university curriculum.

To conclude attendance at social settings can facilitate widespread SARS-CoV-2 transmission in university students. Constraint of transmission in higher education settings needs to emphasise risks outside university premises, as well as a COVID-safe environment within university premises.

## Introduction

The current dominant strain, across Europe, is the omicron BA.5 variant of severe acute respiratory syndrome coronavirus 2 (SARS-CoV-2) [1], with a new influenza season starting it is important to understand the possible sites of disease transmission. In particular universities have been identified as sites where SARS-CoV-2 transmission can readily occur. Along with other sectors of the economy, social distancing and other non-pharmaceutical interventions were mandated by the UK government in higher education during the coronavirus pandemic [2]. These measures were informed by disease transmission modelling [3] and by experiences gained earlier in the pandemic [4].

The University of Cambridge (UoC) used the guidelines outlined by the UK government to create its own set of guidelines that could be implemented in its 31 constituent colleges. Each of these colleges range in geographical size, and in the demographic of their annual intakes. It is in these colleges that students reside, undertake most if not all of their in-person teaching, and attend college-led formal and informal social events.

At the point the UoC student year began in October 2020 [5], the B.1.1.7 SARS-CoV-2 variant was transmitting widely in parts of the UK [6]. Students returning to Universities found themselves studying in ‘COVID-safe’ environments featuring many changes on the pre-SARS-CoV-2 regime, included altered housing arrangements with students mixing in small groups (‘bubbles’), the wearing of masks, social distancing during tuition and the use of distance learning approaches. In addition, some universities put in place free voluntary PCR-based screening programmes for students; the UoC was one, offering an asymptomatic screening programme which is described elsewhere [7]. Such programmes complemented the provision of PCR testing for symptomatic individuals by the state, free of charge at the point of use.

Despite the control measures described, outbreaks occurred among students across the UK higher education sector, which includes over 2.3 million students in 160 institutions. The determinants of these outbreaks are still being studied; residence in larger halls of residence has been identified as one risk factor [4], but determinants of successful COVID-safety in higher education settings are still unclear.

We address this by analysing risk factors for SARS-CoV-2 acquisition in one UK university in Cambridge in the period prior to England's second national lockdown (which commenced 5th November 2020). Up to this time, rates among individuals aged under 60 years were generally increasing across England [8] but in Cambridge local authority these case rates were stable [9]. UK Health Security Agency (UKHSA), a statutory body tasked with outbreak surveillance, became aware of increased incidence in a number of UoC Colleges, identified through both the national symptom-derived SARS-CoV-2 testing and through the work of the UoC asymptomatic screening programme which screened asymptomatic students weekly [7, 10]. UKHSA conducted an analytical epidemiological survey into the determinants of SARS-CoV-2 incidence, results of which we present and discuss here.

## Methods

### Participants

In non-COVID-19-pandemic circumstances the majority of UoC students reside in Cambridge City during Michaelmas term 2020. The study population targeted in this study were UoC students residing in Cambridge during the study period. The pandemic situation during the study period meant not all students out of the possible 25 256 student population, who were sent the questionnaire, were residing in Cambridge at that time. We identified the study population from the 25 256 student group as part of the questionnaire sent to all students: residency was determined through a student's response to the initial survey question. Students who answered as not residing in Cambridge City during this period were taken to the finishing page of the survey and excluded from analytical epidemiological studies.

### Survey

Cases were defined as individuals testing positive for SARS-CoV-2, with or without symptoms, between 30th October 2020 and the date of questionnaire completion, which was 25th or 26th November in almost all cases (see below). All other respondents were considered controls; this included individuals with SARS-CoV-2 like symptoms who either had no test result or a non-positive test result.

Risk factors assessed by the questionnaire administered (included in Supplementary Materials) included: age, gender, ethnicity, UoC college, student type (undergraduate or postgraduate), symptoms of coronavirus disease 2019 (COVID-19) and SARS-CoV-2 test results, term time accommodation, food shopping habits, travel habits and in-person teaching settings. We also asked about attendance, queueing and social distancing at social events attended between Friday 30th October and Wednesday 4th November 2020. We focused on these exposures because of anecdotal observations by colleagues that a number of affected students may have visited such venues.

It was decided to conduct the questionnaire on a de-identified (unnamed) basis in order to encourage full and honest responses from students. Thus, results of tests are self-reported. While the questionnaire asked about attendance at a defined set of named social settings and venues for event attendance, the identities of these have been anonymised as ‘social setting’ or ‘SS’ followed by a number.

The study population were contacted by email with a link to the online questionnaire, which was hosted in Snap Survey, a commercial questionnaire software, on 25th November 2020. The questionnaire was live for one week before it closed to responses at 12:00pm on 3rd December 2020.

### Data analysis

#### Descriptive analysis

Age, gender, ethnicity, UoC college and symptoms of cases and non-cases were described.

#### Data cleaning

Prior to inferential analysis, we generated numerical fields created from open-ended text fields describing other exposures not listed in the questionnaire whereby those individuals not explicitly mentioning the exposure had their values changed from missing to ‘No’. This applied to the fields of ‘college catering’, ‘attended labs’, ‘attended seminar(s)’, ‘medical student placement’ and ‘met in other accommodation’. All other data entries were analysed as entered.

Mixed effects logistic regression was used with the binary response (SARS-CoV-2 positive/negative) as the outcome and student college as the random effect to allow for possible non-independence between student outcomes. The odds ratio (OR) as the measure of effect was used and it, together with its 95% confidence interval (CI), are quoted in the results. The *P* values were obtained by means of the likelihood ratio test or, if not possible, the Wald test. A statistical significance level of *P* ⩽ 0.05 was chosen.

The analysis began by conducting a univariate analysis. This involved fitting a series of models, each with just one fixed effect without regards to other explanatory variable and considering each factor in turn. Those variables with *P* value of 0.2 or less, odds ratio larger than 1.0 and the variables of queueing and social distancing at events were then considered further in a multivariable model in a backwards stepwise procedure wherein, at each step, a fixed effect with the most missing values among those not considered by that stage and *P* value larger than 0.1 was considered for removal from the model. It was removed if it was not substantially confounding. A variable was considered to be substantially confounding if its removal resulted in a change of 10% or more in one or more of the odds ratios for the variables still in the model. The process concluded with the final model when each of the variables in the model met one or more of the following: had been found to be substantially confounding in the one of the preceding steps, had a *P* value of 0.1 or less, or removal would not increase the number of available observations with which to perform a complete-case analysis. The adequacy of excluding the variables dropped during the model building process was checked by adding them one at a time to the final model (and removing it before adding another) to see that each remained non-significant and was not substantially confounding.

For the continuous variable in the dataset (age), a stepwise procedure was performed by beginning with a cubic function (on the logit scale) and simplifying to the next simplest function if the deterioration in fit was not statistically significant until either the function was linear or the least complex function not fitting significantly worse. This was done in the single variable analysis as well as the first step in the multivariable modelling procedure. After this first step, implausible protective factors were removed one at a time until there were no such fixed effects. The subsequent multivariable modelling steps followed the process described above.

All analyses were performed in Stata (StataCorp) versions 15 and 16.1.

## Results

### Cohort studied

**Fig. 1.**
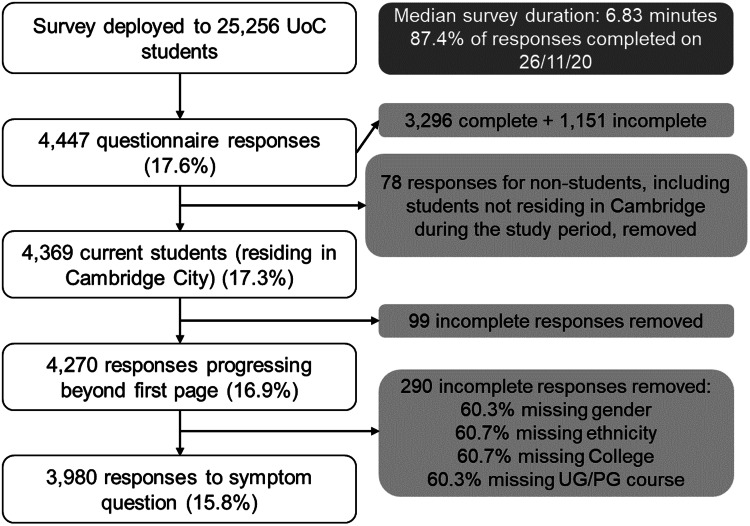
Questionnaire responses, UoC cohort.

The online questionnaire was deployed to a total of 25 256 UoC students. In total 4447 questionnaires were returned, which contained 1151 incomplete responses giving a response rate of 17.6%. We excluded 78 ineligible responses from individuals other than students, and 389 incomplete responses without details of symptoms or key demographic data, leaving 3980 responses in the final analysis (15.8% analysable rate) (Fig. 1).

Out of the 3980 responses used in the final analysis, 99 individuals met the case definition (2.5%) of a positive individual test result for SARS-CoV-2 on or after 30th October 2020, while 3617 individuals did not (264 individuals were unable to be categorised as either – they did not answer any specimen questions). As expected, the reported durations of illness in these individuals matched a spike in incidence reported in Cambridge local authority between 8th and 12th November 2021 [9], detected by national surveillance systems (see Supplementary Fig. S1).

**Fig. 2.**
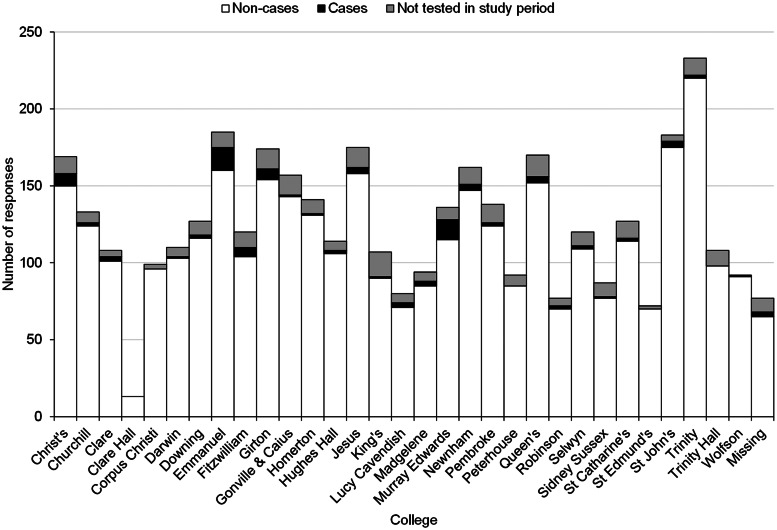
Distribution of cases and non-cases among UoC colleges (*n* = 3980).

**Fig. 3.**
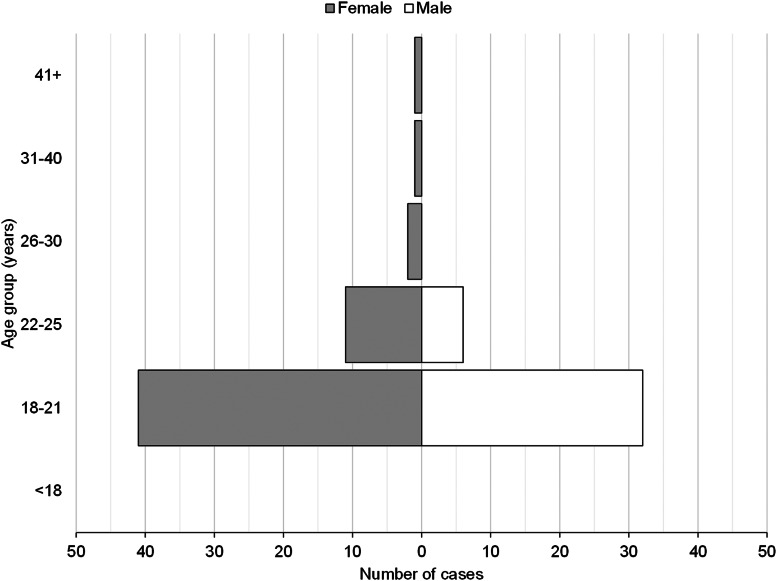
Age and gender distribution among cases, UoC cohort (*n* = 94).

Responses, and positive cases, were received from across Cambridge's colleges (Fig. 2, Appendix 1). The demographic details of respondents is typical of Cambridge students (Fig. 3); the median age was 20 years, with respondents being predominantly white (2935, 74%), while 60% (2386) were undergraduate students (2386) (Table 1, Fig. 3). For more demographic details, see Appendix 2.

**Table 1.** Characteristics of cases and non-cases, UoC cohort (*n* = 3980)

Characteristic	Overall		Cases		Non-cases	
Gender						
Male	1666	41.9%	38	38.4%	1522	42.1%
Female	2193	55.1%	57	57.6%	1989	55.0%
Other	43	1.1%	0	0.0%	41	1.1%
Missing	78	2.0%	4	4.0%	65	1.8%
Age (years)						
Median	21		20		21	
Range	16–50		18–42		16–50	
Ethnicity						
White	2935	73.7%	84	84.8%	2659	73.5%
Indian	130	3.3%	3	3.0%	120	3.3%
Pakistani	25	0.6%	0	0.0%	21	0.6%
Other Asian/Asian British	457	11.5%	2	2.0%	431	11.9%
Black	44	1.1%	1	1.0%	38	1.1%
Mixed	172	4.3%	6	6.1%	154	4.3%
Other	73	1.8%	2	2.0%	68	1.9%
Missing	144	3.6%	1	1.0%	126	3.5%
Student type						
Undergraduate	2386	59.9%	78	78.8%	2108	58.3%
Postgraduate	1581	39.7%	20	20.2%	1499	41.4%
Missing	13	0.3%	1	1.0%	10	0.3%

### Individuals testing positive

99 individuals reported testing positive. The majority of cases reported that their positive result was part of the UoC screening programme (66.7%), with smaller proportions detected by NHS testing (21.2%) and the Cambridge University Hospital screening programme (11.1%). Nearly half of cases (45.5%) reported having had face to face contact with another known case of COVID-19 since 16th October 2020, compared to 9.8% of non-cases.

**Fig. 4.**
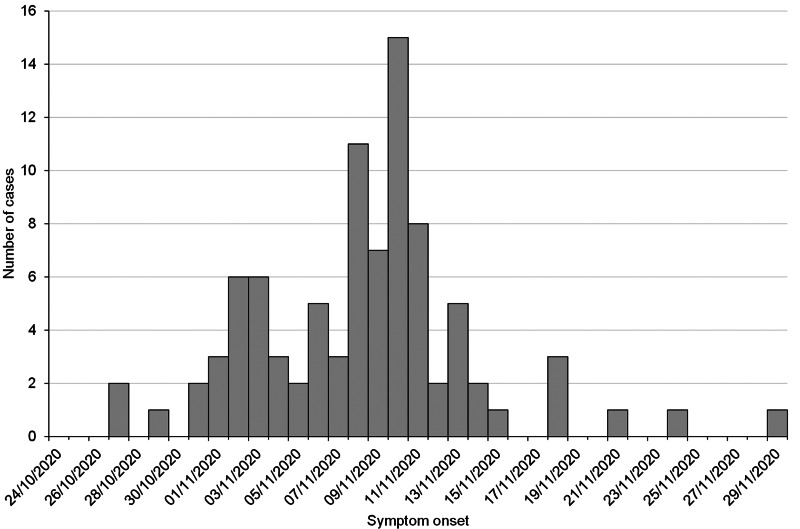
Distribution of cases by date of symptom onset reported, UoC cohort (*n* = 90).

**Fig. 5.**
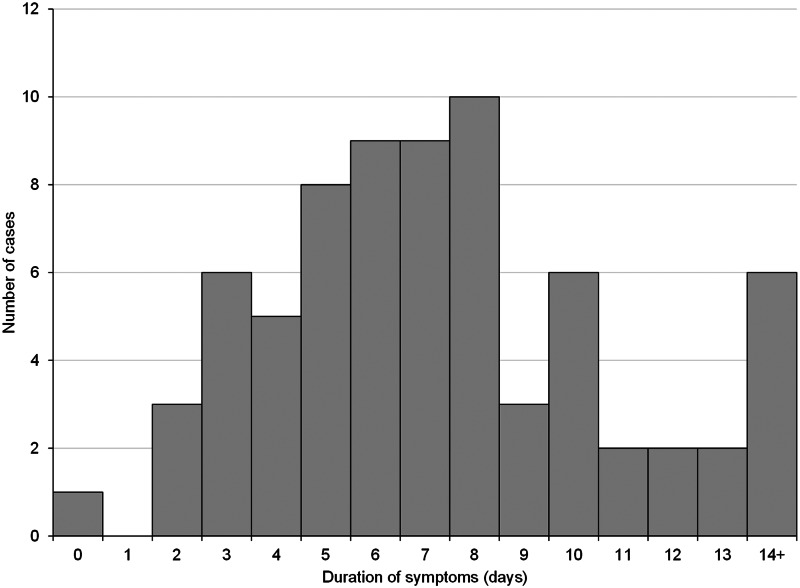
Distribution of the duration of symptoms among cases, UoC cohort (*n* = 72).

The earliest date of symptom onset was 27th October, with the majority of cases reporting onset after the start of national lockdown on 5th November, peaking at 15 cases with onset on 10th November (Fig. 4). Table 2 shows the frequency of symptoms reported among cases, of which the most commonly reported symptoms were COVID-like illness (77.8%) of fever or cough or loss/change of sense of taste/smell, headache (76.8%), sore throat (67.7%), fatigue (64.6%) and runny nose (61.6%). More than one quarter of cases were asymptomatic (28.3%). The median duration of illness was 7 days, ranging from 0 to 38 days, with the full distribution shown in Figure 5. The majority of cases (60) recovered within ten days of symptom onset. For all symptoms except vomiting, the frequency of self-reported symptoms in cases was much higher than in non-cases (Table 2). Details of healthcare consultations following COVID-19 diagnosis are in Table 3.

**Table 2.** Frequency of symptoms among cases and non-cases, UoC cohort (*n* = 3980)

Symptoms	Cases		Non-cases	
Asymptomatic	28	28.3%	–	–
COVID-like illness^a^	77	77.8%	214	5.9%
Fever	46	46.5%	115	3.2%
Cough	34	34.3%	105	2.9%
Loss or change of sense of taste or smell	54	54.5%	65	1.8%
Shortness of breath	42	42.4%	129	3.6%
Sore throat	67	67.7%	644	17.8%
Runny nose	61	61.6%	810	22.4%
Headache	76	76.8%	951	26.3%
Muscle aches	54	54.5%	308	8.5%
Fatigue	64	64.6%	398	11.0%
Diarrhoea	13	13.1%	247	6.8%
Vomiting	2	2.0%	64	1.8%
Other symptoms	18	18.2%	43	1.2%

a Fever or cough or loss/change of sense of taste/smell.

**Table 3.** Type of healthcare consulted by cases, UoC cohort (*n* = 91)

Type of healthcare consulted	Cases	
College nurse	17	18.7%
NHS 111	5	5.5%
GP or another doctor	3	3.3%
Visited A&E	0	0.0%
Other healthcare	2	2.2%
Admitted to hospital	0	0.0%

### Risk factors for SARS-CoV-2 test positivity

In univariate analyses, we observed strong associations between testing positive and attendance at some social settings (most notably attendance at SS7 or SS23). An association with SS3 (representing attendance at Formal Hall) was also noted (OR 2.73, 95% CI 1.2–6.4). Depending on the college, formal hall is an all college weekly tradition where attendees dine together in a communal dining hall. Attendees are seated by academic rank with senior academics dining at a ‘high’ or separated table. Other university associated activities (Appendix 3, Teaching section, as well as Social Settings 14,6 and 35, which were university related) were not significantly associated with disease. In univariate analyses, we also noted disease association with being a postgraduate *vs.* undergraduate, and with type of accommodation (see Table 4 for variables subsequently included the multivariable analysis, and Appendix 3 for all other exposures).

**Table 4.** Single variable analysis of demographics, lifestyle and social event exposures among cases and non-cases, UoC cohort

Variable	Category or measure	Cases	Non-cases	OR	95% CI	*P* value
Demographics						
Age (years)	Minimum	18	16	Quadratic		**0.048**
	25th centile	19	19			
	Median	20	21			
	75th centile	21	24			
	Maximum	42	50			
Gender	Male	38	1522	1.00		**0**.**13**
	Female	57	1989	0.97	0.62–1.51	
	Other	0	41	0.00	n.e.	
Ethnicity	White	84	2659	1.00		**0**.**008**
	Indian	3	120	0.79	0.24–2.57	
	Pakistani	0	21	0.00	n.e.	
	Other Asian/Asian British	2	431	0.14	0.04–0.59	
	Black	1	38	0.88	0.12–6.61	
	Mixed	6	154	1.30	0.55–3.05	
	Other	2	68	0.94	0.23–3.98	
Term time accommodation						
Accommodation type	College flat	7	379	1.00		**<0**.**001**
	College House	19	653	1.47	0.59–3.65	
	College staircase/block	65	1594	2.29	1.02–5.14	
	Private flat	4	303	0.71	0.20–2.47	
	Private house	3	486	0.33	0.09–1.32	
Shared kitchen	Yes	95	3124	2.67	0.83–8.56	**0**.**054**
	No	3	286	1.00		
Food shopping habits						
Go to supermarket	Yes	94	3142	2.58	1.04–6.42	**0**.**02**
	No	5	475	1.00		
Term time travel						
Walk	Yes	83	2776	1.53	0.88–2.65	**0**.**11**
	No	16	841	1.00		
Teaching						
Attended other teaching	Yes	7	121	1.88	0.80–4.46	**0**.**18**
	No	91	3288	1.00		
Event attendance between Friday 30 October and Wednesday 4 November 2020						
Queueing at social events	Did not attend	35	1247	1.00		**0**.**03**
	No queue	14	625	0.83	0.44–1.56	
	Queue only outside	23	369	2.13	1.21–3.73	
	Queue only inside	2	124	0.50	0.18–2.16	
	Queue outside and inside	4	63	1.70	0.56–5.10	
Social distancing at social events	Did not attend	35	1247	1.00		**0**.**2**
	All/most of time	10	490	0.64	0.30–1.34	
	Some of time	26	613	1.52	0.90. 2.57	
	Mixture some/none of time	10	284	1.04	0.49–2.21	
	None of time	10	262	1.31	0.64–2.72	
Student type	Postgraduate	20	1499	2.73	1.62–4.59	**<0**.**001**
	Undergraduate	78	2108	1.00		
Social setting 3	Yes	7	78	2.73	1.17–6.41	**0**.**04**
	No	90	3314	1.00		
Social setting 4	Yes	30	805	1.35	0.86–2.13	**0**.**20**
	No	67	2587	1.00		
Social setting 7	Yes	18	69	13.3	7.21–24.6	**<0**.**001**
	No	79	3323	1.00		
Social setting 17	Yes	2	24	3.77	0.83–17.1	**0**.**14**
	No	95	3368	1.00		
Social setting 21	Yes	2	6	11.2	1.92–65.2	**0**.**02**
	No	95	3386	1.00		
Social setting 22	Yes	6	59	3.86	1.55–9.59	**0**.**01**
	No	91	3333	1.00		
Social setting 23	Yes	3	14	9.45	2.48–35.9	**0**.**007**
	No	94	3378	1.00		
Social setting 30	Yes	5	47	4.04	1.50–10.9	**0**.**02**
	No	92	3345	1.00		

n.e., not estimable.

### Multivariable analysis

Following univariate analysis, nineteen variables were considered in the multivariable model (see Methods for selection criteria, and Table 5). We removed terms making minimal contributions to the model (see Methods), eliminating the terms ‘*go to supermarket*’, ‘*shared kitchen*’, ‘gender’, ‘*other teaching*’, ‘*social setting (SS) 4*’ ‘*SS21*’, ‘*SS22*’, ‘*walking*’ and ‘*student type*’. We also removed the variable on *Queuing at social events*. We did this because it is possible that outdoor queueing is a marker of COVID-19 safe environments to which access is restricted, making interpretation difficult without stratification by venue, something we explore further below. The final model is shown in Table 6.

**Table 5.** Multivariable model (n = 2252), UoC cohort

Variable	Category or measure	OR	95% CI	*P* value
Age	Per year	0.94	0.84–1.04	0.5
Ethnic group	White	1.00		0.02
	Indian	1.01	0.23–4.51	
	Pakistani	0.00	n.e.	
	Other Asian/Asian British	0.10	0.01–0.77	
	Black	0.00	n.e.	
	Mixed	1.34	0.44–4.06	
	Other	1.09	0.14–8.61	
Accommodation type	College flat	1.00		0.6
	College House	1.11	0.40–3.11	
	College staircase/block	1.29	0.50–3.30	
	Private flat	0.69	0.16–3.01	
	Private house	0.35	0.07–1.91	
Queueing at events	Not attended	1.00		0.8
	No queueing	0.71	0.26–1.97	
	Queueing only outside	0.60	0.20–1.83	
	Queueing only inside	0.38	0.07–2.17	
	Queueing inside and outside	0.41	0.09–1.89	
Social distancing (s.d.) at events	Not attended	1.00		0.15
	s.d. all/most of time	0.40	0.12–1.39	
	s.d. some of time	1.17	0.45–3.08	
	Mixture of s.d. some/none of time	0.57	0.17–1.87	
	s.d. none of time	n.e.	n.e.	
Social setting 3	Yes No	3.03 1.00	0.96–9.53	0.08
Social setting 7	Yes No	13.9 1.00	5.52–57.2	<0.001
Social setting 17	Yes No	10.0 1.00	1.76–57.2	0.09
Social setting 23	Yes No	17.3 1.00	3.01–99.4	0.004
Social setting 30	Yes No	3.81 1.00	1.02–14.2	0.06

n.e., not estimable.

**Table 6.** Final multivariable model without ‘queueing at social events’ (*n* = 2825), UoC cohort

Variable	Category or measure	OR	95% CI	*P* value
Age	Per year	0.96	0.87–1.05	0.4
Ethnic group	White	1		0.03
	Indian	0.94	0.27–3.21	
	Pakistani	0	n.e.	
	Other Asian/Asian British	0.18	0.04–0.76	
	Black	0.91	0.11–7.17	
	Mixed	1.84	0.75–4.55	
	Other	1.57	0.35–6.99	
Accommodation type	College flat	1		0.3
	College House	1.33	0.51–3.46	
	College staircase/block	1.56	0.65–3.77	
	Private flat	0.88	0.24–3.29	
	Private house	0.46	0.11–1.93	
Social distancing (s.d.) at events	Not attended	1		0.01
	s.d. all/most of time	0.32	0.14–0.75	
	s.d. some of time	0.7	0.38–1.31	
	Mixture of s.d. some/none of time	0.37	0.15–0.89	
	s.d. none of time	0.64	0.29–1.43	
Social setting 3	Yes	3.1	1.22–7.90	0.02
	No	1		
Social setting 7	Yes	13	6.25–26.9	<0.001
	No	1		
Social setting 17	Yes	7.73	1.54–38.8	0.04
	No	1		
Social setting 23	Yes	14.2	2.90–69.9	0.005
	No	1		
Social setting 30	Yes	2.78	0.85–9.10	0.11
	No	1		

n.e., not estimable.

In the final multivariable model (Table 6) and univariable analysis (Table 5) both the strongest independent associations with positivity were attendance at SS7 (unadjusted OR 13.9 (95% CI 5.52–57.2); adjusted OR (aOR) 13.0 (6.25–26.9)) and SS23 (OR 17.3 (95% CI 3.01–99.4); aOR 14.2 (2.90–69.9)). There is also some evidence of independent association with positivity, of attendance at social settings SS3 (Formal Hall), and with SS17 and SS30, which are commercial venues at which socialisation occurred. The strength of the observed effect differed slightly depending on the model used (Table 5 *vs.* Table 6).

Interestingly, of the students that attended SS7 and answered questions about queuing and social distancing (*n* = 68), there were variable reports about the extent of social distancing, with 33 respondents stating that social distancing was practiced all the time and 39 responding that it was practiced some of the time. In SS23, 17 of the respondents reported social distancing being practiced all (8 responses), or some of the time (5 responses). See Appendix 4 for full tabulation.

It is notable that neither undergraduate/postgraduate status, nor attendance at university teaching form part of the final model, and that the contribution of accommodation type is not significant. It appears that the associations of SARS-CoV2 acquisition with these risk factors are captured by other risk factors, notably the attendance at social events.

## Discussion

This investigation has found strong evidence of independent association with SARS-CoV-2 detection and attendance at two social venues, with weaker evidence at others. The highest odds were with attendance at SS7 and SS23 (aOR 13.9 (95% CI 5.5–57) and aOR 17.3 (3–99) respectively, both were primarily settings where food and drink were served and consumed indoors. This is also a feature of SS3 (Formal Hall) which was similarly, but more weakly, associated with odds of disease (aOR 3.0, 95% CI 0.96–9.5). Such indoor settings are recognised to represent a risk [11, 12]. In the time period of this study the wearing of face coverings in certain indoors setting was a mandatory legal requirement [13] unless sat at an assigned table. However, it would be expected, due to the consumption of food and beverages, that constant wearing of a face covering or mask would be difficult to maintain; adding to this point, respondents indicated inconstant social distancing. In contrast, it was notable that neither university attendance, type of residence, or student category contributed significantly to odds of positivity, and neither did attendance at the majority of university organised or based settings (SS1, SS2, SS4, SS6 and SS35). Responses received, and the timing of illness, suggests that socialising between students in non-university settings occurred shortly before a national lockdown was imposed, and in the context of a rapidly spreading SARS-CoV-2 epidemic. Our work also suggests that the control measures put in place by the university [14] were largely effective at minimising the odds of infection, with the possible exception of Formal Hall related dining.

This research study has several notable limitations. Firstly, by design it was anonymous, and so the responses obtained could not be checked against national information systems. Secondly, the survey was retrospective, so attendance at events or recording of symptoms may be subject to recall bias. Finally, we received 3980 responses to the questionnaire from a potential study population of 25 256, a response rate of 15.8%. While there is evidence of external validity of the responses obtained – in particular, the time course of the development of illness reported matches what actually happened – the low response rates mean that the conclusions require external validation. Such external validation has recently been published, in the form of a genomic analysis of sequences from individuals with SARS-CoV-2 infection [10], which also strongly implicates social mixing outside of university settings as a key risk factor for SARS-CoV-2 infection. Further external validation can be seen through comparison of the cases reported in Cambridge local authority [9], and those by specimen date reported by the UoC cohort (see Supplementary Fig. S1). The cases in both figures peak around the same time period (8th – 12th November 2021).

This work builds on studies from elsewhere identifying indoor social settings as sites of SARS-CoV-2 transmission [12]. For example, at the start of the pandemic, nightclubs in Seoul reported multiple cases associated with venues of this type [15], and since the easing of social distancing and lockdown measures in South Korea, nightclubs have been highlighted as venues of concern, where cases could easily spread to the wider community [16]. Our results indicate that, in university settings, infection control measures aimed at establishing a low transmission ‘COVID-safe’ learning environment can readily be compromised by attendance at social gatherings – an aspect for consideration as Universities across Europe commence new academic years come Autumn.

## Data Availability

The data that support the findings of this study are available on request from the corresponding author, EB. The data are not publicly available due to their containing information that could compromise the privacy of research participants.

## Supplementary material

The supplementary material for this article can be found at https://doi.org/10.1017/S0950268822001698
